# Overexpression of GLT1D1 induces immunosuppression through glycosylation of PD‐L1 and predicts poor prognosis in B‐cell lymphoma

**DOI:** 10.1002/1878-0261.12664

**Published:** 2020-04-13

**Authors:** Xiaoxia Liu, Yanyu Zhang, Yi Han, Wenhua Lu, Jing Yang, Jingyu Tian, Peng Sun, Tiantian Yu, Yumin Hu, Hui Zhang, Peng Huang, Panpan Liu

**Affiliations:** ^1^ State Key Laboratory of Oncology in Southern China Collaborative Innovation Center for Cancer Medicine Sun Yat‐Sen University Cancer Center Guangzhou China; ^2^ The Sixth Affiliated Hospital Sun Yat‐sen University Guangzhou China; ^3^ Metabolic Innovation Center Sun Yat‐Sen University Guangzhou China; ^4^ Department of Medical Oncology Sun Yat‐Sen University Cancer Center Guangzhou China

**Keywords:** B‐cell non‐Hodgkin's lymphoma, GLT1D1, glycosylated PD‐L1, immunosuppression, prognosis

## Abstract

B‐cell non‐Hodgkin's lymphoma (NHL) is a class of heterogeneous diseases with variable clinical outcomes. Immunosuppression is particularly common in the subtypes of lymphoma with poor prognosis, but the underlying mechanism remains unclear. Using a RT‐PCR array analysis, we have identified that glycosyltransferase 1 domain‐containing 1 (GLT1D1), an enzyme that transfers glycosyl groups to proteins, is highly upregulated in the incurable subtype of B‐cell NHL and in early relapse diffuse large B‐cell lymphoma. Analysis of clinical specimens revealed that GLT1D1 expression was positively correlated with the level of glycosylated programmed cell death‐ligand 1 (PD‐L1) in B‐cell NHL and that high GLT1D1 expression was associated with poor prognosis. Mechanistically, we showed that GLT1D1 transferred N‐linked glycans to PD‐L1, thus promoting the immunosuppressive function of glycosylated PD‐L1. Downregulation of GLT1D1 resulted in a decrease of glycosylated PD‐L1 and enhanced cytotoxic T‐cell function against lymphoma cells. *In vivo*, overexpression of GLT1D1 promoted tumor growth by facilitating tumor immune escape through increased levels of PD‐L1. Our work has identified GLT1D1 as a predictive biomarker for B‐cell NHL. It has also shown that this enzyme enhances PD‐L1 stabilization via N‐glycosylation, thus promoting immunosuppression and tumor growth. As such, GLT1D1 might be a novel therapeutic target for the treatment of B‐NHL.

## Graphical abstract

B‐cell non‐Hodgkin's lymphoma (B‐NHL) is a heterogeneous group of diseases with different biological characteristics and clinical outcomes. The expression of glycosyltransferase 1 domain‐containing 1 (GLT1D1) in B‐NHL was positively correlated with the level of glycosylated PD‐L1, and high GLT1D1 expression was associated with poor prognosis. GLT1D1 transferred N‐glycans to the asparagine residue of PD‐L1 and thus enhances its stability, leading to immunosuppression and poor clinical outcome.

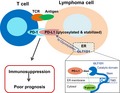

**Abbreviations**
ASCT: autologous stem cell transplantation
BL: Burkitt lymphoma
DLBCL: diffuse large B‐cell lymphoma
FL: follicular lymphoma
GEO: Gene Expression Omnibus
GLT1D1: glycosyltransferase 1 domain‐containing 1
HL: Hodgkin lymphoma
MCL: mantle cell lymphoma
NHL: non‐Hodgkin's lymphoma
OS: overall survival
PD‐1: programmed death‐1
PD‐L1: programmed death‐ligand 1
PFS: progression‐free survival
TM: tunicamycin

## 1 Introduction

Non‐Hodgkin's lymphoma (NHL) is a cancer originated in the lymphatic system, characterized by the neoplastic transformation and abnormal proliferation of lymphocytes. NHL is among the ten most commonly diagnosed cancers both in Western countries and in Asia. Pathologically, NHLs are a heterogeneous group of neoplasms mostly arise from B cells. Subtypes of NHLs that involve B cells include diffuse large B‐cell lymphoma (DLBCL), follicular lymphoma, mantle cell lymphoma (MCL), Burkitt lymphoma (BL), and others. Patients with NHLs who experience a relapse after initial treatment or autologous stem cell transplantation (ASCT) usually have limited treatment options and generally have a poor prognosis (Friedberg, 2011; Gisselbrecht *et al.*, 2010; Teras *et al.*, 2016; Van Den Neste *et al.*, 2017). DLBCL is the most common subtype of NHL, and about 2/3 of the patients could be cured by standard front‐line therapy. However, patients with refractory DLBCL or those who are unsuitable for or who have experienced relapse after ASCT have limited treatment options, with a median survival of only 6–10 months from progression (Crump *et al.*, 2017; Nagle *et al.*, 2013). BL is a highly aggressive B‐cell neoplasm characterized by the translocation and deregulation of the c‐Myc gene on chromosome 8. A majority of patients with BL may be cured with aggressive treatment regimens (Dunleavy *et al.*, 2013; Mead *et al.*, 2008; Thomas *et al.*, 1999). Patients with refractory disease and those who relapse after an initial response to appropriate front‐line therapy have a very poor prognosis due to resistance to subsequent treatment. MCL is an uncommon subtype of B‐cell NHL that accounts for 5–10% of all lymphomas. MCL is generally incurable with a median overall survival (OS) of only 4–5 years and is considered a disease with poor prognosis (Cohen *et al.*, 2017; Teras *et al.*, 2016). Understanding the biochemical features of relapsed or refractory NHLs and developing more effective therapies are needed to further improve NHL outcome.

The programmed death‐1/programmed death‐ligand1 (PD‐1/PD‐L1) pathway is an important co‐inhibitory pathway to attenuate T cell‐mediated immune responses and may be exploited by tumors to avoid immune surveillance (Chen *et al.*, 2013). PD‐1 and its ligands PD‐L1 and PD‐L2 are often expressed in tumor or neoplastic microenvironments, including epithelial malignancies, classical Hodgkin lymphoma (HL), and NHL, although the expression of PD‐L1 seems to vary significantly among lymphoma subtypes (Andorsky *et al.*, 2011; Chen *et al.*, 2013; Wilcox *et al.*, 2009). Blockade of PD‐1 with nivolumab and pembrolizumab could produce durable objective responses and improve OS in patients with HL (Armand *et al.*, 2018; Chen *et al.*, 2017). However, the efficacy of PD‐1 antibody in NHL is low (Ansell *et al.*, 2019; Ding *et al.*, 2017; Lesokhin *et al.*, 2016), which promoted us to further explore the immunoregulatory mechanisms aiming to find novel strategy to improve response to immunotherapy. Upregulation of PD‐L1 is associated with poor prognosis in patients with DLBCL (Georgiou *et al.*, 2016; Kiyasu *et al.*, 2015). Li *et al* demonstrated that the glycosylation of PD‐L1 can stabilize PD‐L1 protein expression, which is modulated by N‐glycosylation (Li *et al.*, 2016). Furthermore, they showed that patients who had a high N‐glycosyltransferase B3GNT3 (member of the beta‐1,3‐N‐acetylglucosaminyltransferase family) expression also had poorer OS outcomes. B3GNT3 is also known to mediate PD‐L1 glycosylation in triple‐negative breast cancer cells (Li *et al.*, 2018). It remains unknown whether the glycosylation of PD‐L1 in B‐cell NHL is regulated by B3GNT3 or other glycosyltransferases.

In this study, we developed a N‐linked glycosylation qRT‐PCR array to screen 9 B‐cell NHL cell lines representing three subtypes of NHLs. The result showed that glycosyltransferase 1 domain‐containing protein 1 (GLT1D1) was upregulated in the incurable MCL. Subsequent analysis of clinical data revealed that high expression of GLT1D1 was correlated with poor prognosis of DLBCL patients. We further demonstrated that GLT1D1 could promote the N‐glycosylation level of PD‐L1 and impair the cytotoxic T‐cell function against tumor cells. Our findings indicated that GLT1D1 could be a novel biomarker for NHLs with poor prognosis due to its ability to stabilize PD‐L1 via N‐glycosylation and thus promote immunosuppression.

## 2 Materials and methods

### 2.1 Reagents and antibodies

Antibody against α‐Tubulin was purchased from Cell Signaling Technology (Danvers, MA, USA). Antibodies against GLT1D1 (mouse) and PD‐L1 were purchased from Abcam (Cambridge, MA, USA). Antibody against human GLT1D1 was purchased from Sigma‐Aldrich (St. Louis, MO, USA). Human GLT1D1 small interfering RNA (siRNA) was purchased from RiboBio (Guangzhou, China).

### 2.2 Cell lines and cell culture

Human BCL cell lines SU‐DHL‐2, SU‐DHL‐4, SU‐DHL‐6, RAJI, Daudi, Namalwa, Jeko‐1, JVM2, and Mino, mouse melanoma cell line B16 and mouse B lymphoma cell line A20 were obtained from the American Type Culture Collection (ATCC, Manassas, VA, USA). Cells were cultured in Roswell Park Memorial Institute (RPMI) 1640 medium (GIBCO, Grand Island, NY, USA) supplemented with FBS (Biological Industries, Kibbutz Beit Haemek, Israel) according to the ATCC manual and incubated in a humidified incubator at 37 °C supplemented with 5% carbon dioxide. For treatment with tunicamycin (TM), cells in the exponential growth phase were seeded in triplicate at a density of 1 × 10^6^ cells·mL^−1^ in 6‐well plates and incubated with TM (1 μg·mL^−1^) for 48 h. The TM‐treated cells were then harvested for further analysis of gene expression and protein glycosylation as indicated in the respective figure legends.

### 2.3 qRT‐PCR

Total cellular RNA was extracted from clinical tumor samples or cell lines (control or siRNA‐treated for 48 h), using the Trizol reagent from Life Technologies (Carlsbad, CA, USA), according to the manufacturer's protocol. The real‐time monitoring of PCR amplification was conducted using the Bio‐Rad Real‐Time PCR System (Life Technologies) and the SYBR Green Realtime Master Mix (TaKaRa, Tokyo, Japan). The primer sequences are listed in Table S1.

### 2.4 Protein extraction and western blot analysis

Cells harvested from culture or isolated from tumor samples were lysed in SDS‐lysis buffer (10 mm Tris‐HCl, pH 6.8, 5% SDS, 10 mm EDTA, 50 mm NaCl), and protein concentrations of the samples were determined using the Pierce™ BCA Protein Assay Kit (Thermo Scientific, Waltham, MA, USA). Protein lysates were analyzed by standard SDS/PAGE and transferred to a polyvinylidene fluoride (PVDF) membrane. Protein bands of interest were revealed by blotting with the respective antibodies. The density of the protein bands was analyzed using the imagej software (Bethesda, MD, USA).

### 2.5 GLT1D1 knockdown with siRNA

siRNAs specifically targeting GLT1D1 and negative controls were purchased from RiboBio (RiboBio, Guangzhou, China). The sequences of siRNA are as follows: st‐h‐GLT1D1_1: GGAAGGGAATACGTGAGAA; st‐h‐GLT1D1_2: GTGGAACTGATGTAAATGA. The GLT1D1 siRNAs were transfected into B lymphoma cells using the Neon Transfection System (Invitrogen, Carlsbad, CA, USA) according to the manufacturer's instructions. Briefly, cells were washed with PBS, resuspended in 100 μL of resuspension buffer (R‐buffer) at a density of 1.0 × 10^7^ cells·mL^−1^, and mixed with 10 μL (20 μm) of GLT1D1 siRNA in a sterile Eppendorf tube. The cells–siRNA mixture was subjected to two pulses with a pulse width of 20 ms at 1400 V. After incubation in culture medium for 72 h, the cells were lysed and analyzed by western blot.

### 2.6 Isolation of effector CD8^+^ T cells from peripheral blood

Mononuclear cells were isolated from peripheral blood samples of healthy adult donors (*n* = 3) using Ficoll‐Paque PLUS (GE Healthcare Bio‐Sciences, Uppsala, Sweden) gradient centrifugation as previously described (Vereide *et al.*, 2014). Peripheral blood mononuclear cells at a density of 1 × 10^7^ per mL were incubated with CD8‐coated magnetic microbeads (Miltenyi Biotec, Bergisch Gladbach, Germany) for 15 min at 4 °C. The cell suspension was loaded onto a magnetic activated cell sorter (MACS) column placed in the magnetic field of a MACS separator. The magnetically labeled CD8^+^ T cells were retained within the column during washing with PBS. The CD8^+^ T cells were then eluted after removing the column from the magnetic field.

### 2.7 Cell co‐culture cytokine measurements

The co‐culture of human effector CD8^+^ T cells and tumor cells and the measurement of IL‐2 and interferon‐gamma (IFN‐γ) expression were performed as previously described (Yang *et al.*, 2017). To analyze the effect of tumor cells on T‐cell inactivation, the tumor cells were co‐cultured with activated effector CD8^+^ T cells, which were stimulated with 2.5 ng·mL^−1^ of phorbol 12‐myristate 13‐acetate (PMA) plus 500 ng·mL^−1^ of ionomycin (Bueno *et al.*, 2002). CD8^+^ T cells and tumor cells were co‐cultured at a 5 : 1 ratio for 48 h. The culture medium was collected, and the secreted IL‐2 and IFN‐γ in the medium were measured using ELISA kits according to the manufacturer's instructions (Human IL‐2 ELISA Kits, Abcam; Human IFN‐γ ELISA Kits; eBiosience, San Diego, CA, USA).

### 2.8 Assay for T cell‐mediated cytotoxicity

Activated human effector T cells and tumor cells (5 : 1) were co‐cultured for approximately 72 h. After the gravity sedimentation of microcarriers and detachment of cells, the lactate dehydrogenase (LDH) activity in the culture supernatants was analyzed by mixing the cell culture supernatant (100 μL) with 100 μL of LDH assay reagent (BioVision, Milpitas, CA, USA) according to the manufacturer instructions. The changes in absorbance at 492 nm were read for up to 30 min at room temperature. LDH activity values are shown as OD values. Cellular injury was evaluated by measuring the release of LDH.

### 2.9 Glycoprotein deglycosylation

To determine the level of PD‐L1 protein glycosylation, the deglycosylation of PD‐L1 protein was performed using the enzymatic protein deglycosylation kit from Sigma (St. Louis, MO, USA), and assay of GLT1D1 deglycosylation was performed using a PNGase F digestion kit (NEB, New England Biolabs, Beverly, MA, USA) according to the manufacturer instructions. The protein extraction reagents did not contain SDS. The protein after deglycosylation was assayed by SDS/PAGE.

### 2.10 Staining of tumor tissues

Tumor tissues were fixed with 4% formaldehyde and embedded in paraffin. The specimens were then cut into 4‐μm sections and stained with hematoxylin and eosin for histopathologic analysis under light microscopy. For immunohistochemistry, the paraffin‐embedded tumor tissue sections were immunostained with monoclonal antibodies against human PD‐L1 or CD8 (Bioss, bs‐4790R) using heat‐mediated antigen retrieval method. Briefly, tissue slides were incubated for 1 h at 60 °C and then washed with tissue cleaner reagent (Hangzhou Sinai Mount Biotech Co. Ltd, Hangzhou, China) for 15 min to remove paraffin. After rehydration and antigen retrieval, the slides were washed and incubated with 3% H_2_O_2_ for 20 min to quench the endogenous peroxidase activity. Then, the slides were washed again with PBS and blocked with 10% goat serum. A monoclonal antibody against human PD‐L1 (1 : 100) or human CD8 (1 : 1000) was added and incubated overnight at 4 °C. Biotinylated secondary antibody was then added and incubated for 1 h at 37 °C. After washing with PBS, the slides were incubated with 3, 3‐diaminobenzidine tetrahydrochloride (DAB) substrate, washed, and examined under a light microscope.

### 2.11 CRISPR‐Cas9‐mediated knockout (GLT1D1) screen

The single‐guide RNA (sgRNA) for GLT1D1 was designed using the optimized CRISPR design protocol (http://crispr.mit.edu/). The production and infection of lentiviruses specifically targeting the GLT1D1 gene were performed as described for the GeCKO library. Two GLT1D1 gene‐targeted and control gRNAs were inserted into the LV3(H1/GFP&Puro) vector (CRISPR/Cas9 knockout plasmid) to construct three recombinant plasmids, GLT1D1‐1, 5′‐GCAACAATACCAAATGCCAAC‐3′; GLT1D1‐2, 5′‐GGAATACGTGAGGACGCATCA‐3′; control, 5′‐TTCTCCGAACGTGTCACGT‐3′. The insertions were confirmed by sequencing. The recombinant plasmids together with lentivirus packaging plasmids were cotransfected into 293T cells to obtain lentivirus particles, and the virus titer was determined. Lymphoma A20 cells were seeded in 60‐mm dishes per replicate at a density of 1 × 10^6^ cells·mL^−1^ in 10 mL of complete medium per dish and infected with lentivirus particles at a multiplicity of infection (MOI) of 0.3 in the presence of polybrene (4 µg·mL^−1^). Three replicate infections per cell line were used. Two days after infection, the A20 cells were selected with puromycin (2 µg·mL^−1^) for 3 days. The cells were then cultured in RPMI 1640 medium without puromycin for 7 days before the subsequent experiments. Similar method was used to infect B16 melanoma cells. Upon reaching 80% confluence, B16 cells were infected with lentivirus particles at a MOI of 0.3 in the presence of polybrene (4 µg·mL^−1^). Two days after infection, B16 cells were selected with puromycin (2.5 µg·mL^−1^) for 2 weeks. Colonies formed in the GLT1D1‐1 and GLT1D1‐2 groups were individually selected and expanded. The protein levels of GLT1D1 and PD‐L1 were detected by western blot.

### 2.12 Patient samples

Clinical specimens from 46 lymphoma patients, who were diagnosed with BCL at Sun Yat‐Sen University Cancer Center during the period from January 2015 to January 2017 with clinical follow‐up data, were included in this study. The use of clinical samples was approved by the ethical committee of Sun Yat‐sen University Cancer Center.

### 2.13 Animal studies

Female C57BL mice aged 5–6 weeks were obtained from Beijing Vital River Laboratory Animal Technology Co. Ltd (Beijing, China). All experimental procedures using these mice were carried out in accordance with a research protocol approved by the Institutional Laboratory Animal Care and Use Committee of Sun Yat‐sen University Cancer Center. Each mouse was inoculated s.c. in the dorsal flank with 2 × 10^6^ B16 cells (control or GLT1D1‐knockout clone 1# or 2#) suspended in 0.1 mL of serum‐free medium. Tumors were measured every 2–3 days, and their volumes were calculated using the following standard formula: (length × width^2^)/2. At the end of the experiment, mice were sacrificed, and tumors were collected, photographed, and weighed.

### 2.14 Database analysis

A publically available database was used to analyze the prognostic significance of GLT1D1 in DLBCL patients. The prognostic analysis was retrieved from the Gene Expression Omnibus (GEO) database. The data set (http://www.ncbi.nlm.nih.gov/geo/query/acc.cgi?acc=GSE10846) comprised 420 cases and was submitted by Lenz *et al.* (2008) and the data set (http://www.ncbi.nlm.nih.gov/geo/query/acc.cgi?acc=GSE23501) comprised 69 cases and was submitted by Shaknovich *et al.* (2010).

### 2.15 Measurement of glycan residues

Glycan residues were analyzed as previously described (Badur *et al.*, 2015). Cells (5 × 10^5^) were rinsed with 0.9% w/v saline and centrifuged at 250–300 ***g*** for 3 min. Collected cell pellets were quenched by 500 μL of −80 °C MeOH. Additional 200 μL of ice‐cold water and 500 μL of −20 °C chloroform were added into the lysates. After vortexing and centrifugation, the top aqueous layer and bottom organic layer were discarded. The interface layer containing biomass was washed twice by −80 °C 500 μL of MeOH and centrifuged at 21 000 ***g***. The interface layers were then dried by ambient air and further hydrolyzed to release glycan residues in 500 μL of 2 m HCl at 80 °C for 2 h. Hydrolyzed biomass solution was dried by nitrogen gas flow for subsequent derivatization. Methoxime‐trimethylsilyl (TMS) derivatives of glycan residues were formed by addition of 15 μL 2% (w/v) methoxyamine hydrochloride (Sigma‐Aldrich) in pyridine and incubated at 37 °C for 60 min. Samples were then silylated by addition of 15 μL of *N*‐methyltrimethylsilyltrifluoroacetamide (MSTFA; Regis Technologies, Morton Grove, IL, USA) and incubated at 45 °C for 30 min. The derivatized products of hydrolyzed interface samples were analyzed by GC/MS using the Thermo Fisher Trace 1300 with the 30m TM‐35ms column (Thermo Fisher, Waltham, MA, USA) connected to the Thermo Fisher ISQ MS QD. GC/MS was operated under electron impact (EI) ionization at 70 eV and using helium as the carrier gas at a flow rate of 1.5 mL·min^−1^. For analysis of hexose derivatives, the sample was injected at 1 µL in splitless mode at 270 °C, and the GC oven temperature was held at 100 °C for 2 min, increased to 255 °C at 3.5 °C·min^−1^, and then ramped to 300 °C at 25 °C·min^−1^ for a total run time of approximately 48 min. The ion source and MS transfer line were held at 300 °C and 250 °C, respectively. The detector was operated in full scanning mode, recording ion abundance in the range of 100–650 *m/z*. The hexoses were identified with standard compounds and further quantified by selected ion fragments *m/z* 319 using Thermo Fisher Xcalibur and TraceFinder 3.3 SP1 GQ (Waltham, MA, USA). Relative abundance of hexose was normalized by total protein of cell samples. The GC/MS was done at the Metabolic Innovation Center of Sun Yat‐Sen University.

### 2.16 Statistical analysis

Data are expressed as the means ± SD and analyzed via prism graphpad 7.0 (GraphPad Software, La Jolla, CA, USA). ANOVA, Student's *t*‐test, or Pearson's correlation analysis was used to measure difference whose significance threshold was set as *P* < 0.05.

## 3 Results

### 3.1 Overexpression of GLT1D1 is associated with poor prognosis in B‐cell NHL

**Fig. 1:**
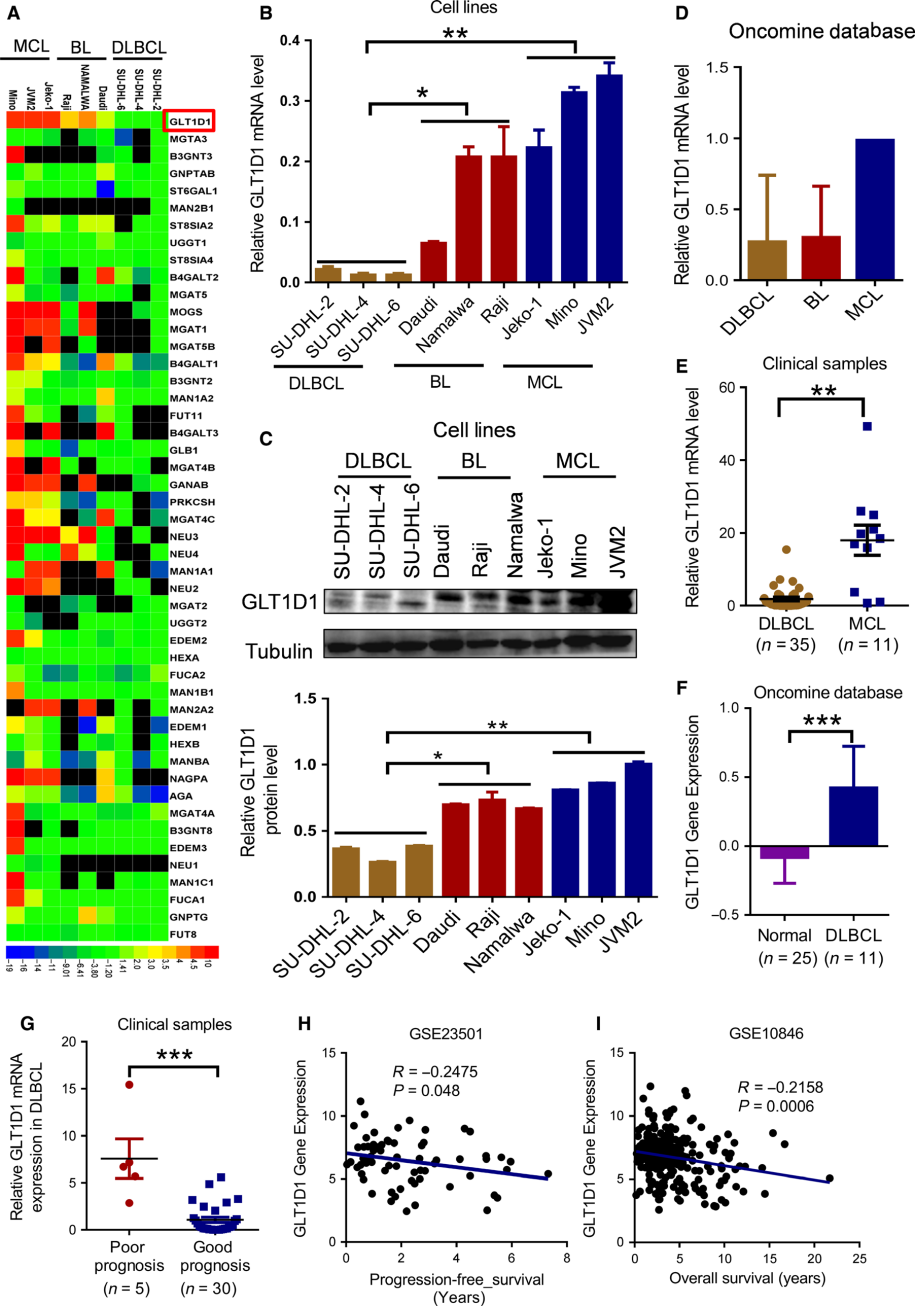
High expression of GLT1D1 is associated with poor prognosis of B‐cell NHLs. (A) Expression of genes encoding N‐glycosyltransferase enzymes in nine B‐cell lymphoma cell lines representing three subtypes of B‐cell NHLs analyzed by qRT‐PCR arrays. The heat map indicates the fold‐change values (in log2 scale) relative to the mean expression levels within the respective cell lines. Red color indicates high expression; blue color indicates low expression. (B) GLT1D1 mRNA expression levels in nine cell lines representing three subtypes of B‐cell NHLs. The bars represent means ± SD of at least three separate measurements by qRT‐PCR (One‐way ANOVA). (C) Comparison of GLT1D1 protein expression nine cell lines representing three subtypes of B‐cell NHLs. A representative western blot is on the upper panel. Quantitative results of three separate assays are shown on the lower panel (one‐way ANOVA). (D) Comparison of GLT1D1 mRNA levels among three subtypes of B‐cell lymphomas (DLBCL, BL, MCL), based on analysis of the Oncomine database (the Wooster B‐cell NHL dataset). (E) GLT1D1 mRNA expression in DLBCL and MCL patient samples. Student's *t*‐test was used. (F) Comparison of GLT1D1 gene expression between normal and DLBCL samples based on analysis of Oncomine database (the Brune Lymphoma dataset). Student's *t*‐test was used to determine the statistical significance. (G) Comparison of GLT1D1 mRNA level in DLBCL patients with good and poor prognosis (Student's *t*‐test). (H) Correlation between expression of GLT1D1 and PFS in DLBCL patients (Pearson's correlation analysis) based on analysis of the http://www.ncbi.nlm.nih.gov/geo/query/acc.cgi?acc=GSE23501 dataset. (I) Correlation between the expression of GLT1D1 and the OS in DLBCL patients based on analysis of http://www.ncbi.nlm.nih.gov/geo/query/acc.cgi?acc=GSE10846 dataset (Pearson's correlation analysis). Bars, means ± SD. *P < 0.05, **P < 0.01, ***P < 0.001.

As high level of PD‐L1 is associated with poor clinical outcome in DLBCL (Chen *et al.*, 2013; Georgiou *et al.*, 2016; Kiyasu *et al.*, 2015), and N‐glycosylated PD‐L1 has been shown to be essential for PD‐L1 stabilization and function (Hsu *et al.*, 2018; Li *et al.*, 2016, 2018), a quantitative real‐time polymerase chain reaction (qRT‐PCR) array containing 50 key genes encoding enzymes that post‐translationally add or remove sugar residues to or from proteoglycans and glycoproteins (Table S1) was used to investigate whether N‐glycosyltransferase changes are involved in regulation of PD‐L1 in B‐cell NHLs. The results revealed that among the genes that were differentially expressed between the highly curable B‐NHLs (DLBCL and BL) and the incurable MCL cells, glycosyltransferase 1 domain‐containing protein 1 (GLT1D1) was highly expressed in the MCL, which has the worst prognosis among the 3 subtypes of B‐cell NHLs (Fig. 1A). We then examined the expression of GLT1D1 in the 3 subtypes of B‐cell NHL cell lines by qRT‐PCR and western blot. The mRNA and protein levels of GLT1D1 were found to be significantly upregulated in the MCL cell lines (Jeko‐1, Mino, JVM2) compared with BL cell lines (Daudi, Raji, Namalwa) (*P* < 0.05) or with DLBCL cell lines (SU‐DHL‐2, SU‐DHL‐4, SU‐DHL‐6) (*P* < 0.01) (Fig. 1B,C). In addition, the high expression of GLT1D1 in MCL was further substantiated by analysis of the Oncomine dataset (Fig. 1D). Importantly, our analysis of clinical samples revealed a significant increase in GLT1D1 mRNA expression in MCL patients (*n* = 11) compared with DLBCL patient samples (*n* = *35*, *P* < 0.01, Fig. 1E).

We then further examined the expression of GLT1D1 expression in DLBCL with good and poor prognosis. DLBCL patients who relapse within 6 months are usually refractory to chemotherapy with an OS of only 4–6 months (defined as poor prognosis group), whereas patients with no recurrence within 1 year after treatment usually have better clinical outcome (good prognosis group). Analysis of Oncomine data revealed an increase of GLT1D1 mRNA expression in the DLBCL cells (*n* = 11) compared with the normal B cells (*n* = 25, *P* < 0.001, Fig. 1F). The DLBCL patients with poor prognosis (*n* = 5) had significant upregulation of GLT1D1 compared to those with good prognosis (*n* = 30) (*P* < 0.001) (Fig. 1G), suggesting that overexpression of GLT1D1 is associated with poor prognosis in lymphoma. To further confirm this relationship, we analyzed GLT1D1 expression and the survival probability of DLBCL from the GEO database. The correlation between GLT1D1 expression and progression‐free survival (PFS) and OS were analyzed. A statistically significant negative correlation was detected between GLT1D1 expression and the PFS of patients in the http://www.ncbi.nlm.nih.gov/geo/query/acc.cgi?acc=GSE23501 dataset (Fig. 1H), and between GLT1D1 expression and OS of patients in the http://www.ncbi.nlm.nih.gov/geo/query/acc.cgi?acc=GSE10846 dataset (Fig. 1I).

### 3.2 GLT1D1 catalyzes N‐glycosylation of PD‐L1 in B‐cell NHL

**Fig. 2:**
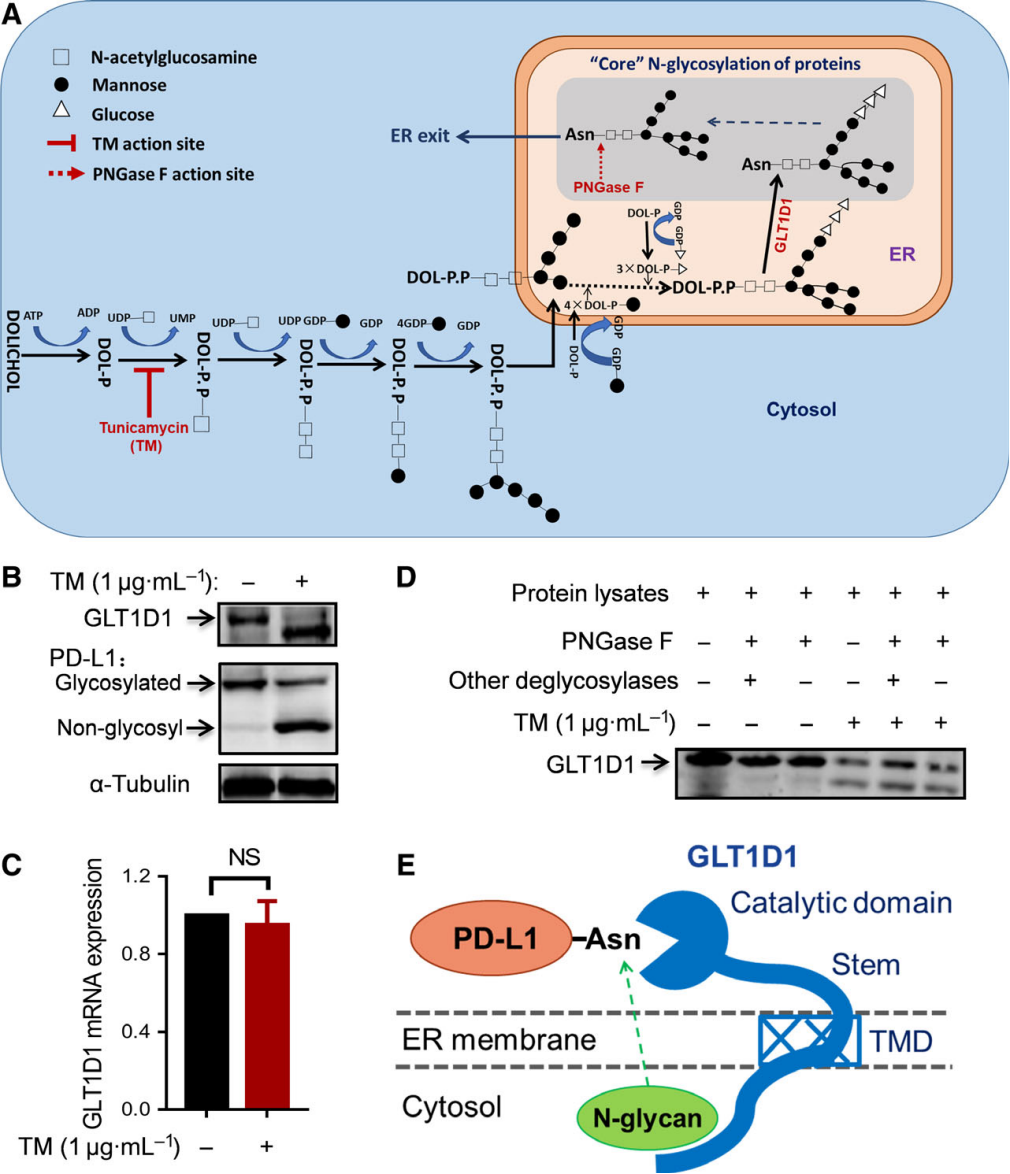
Impact of tunicamycin and deglycosylases on GLT1D1 expression and PD‐L1 glycosylation. (A) Schematic illustration of protein N‐glycosylation pathway. Synthesis of oligosaccharide starts on the cytosolic surface of the ER membrane by the addition of sugars to dolichylphosphate. The oligosaccharide is then flipped to the lumen side of the ER membrane, where a 14‐saccharide core unit is assembled as a membrane‐bound dolichylpyrophosphate (DOL‐PP), which is further processed in the ER lumen for transferring to the nascent polypeptide chains. GLT1D1 functions as glycosyltransferase to transfer N‐glycans to the asparagine (Asn) residues of the target polypeptides. The action sites of TM and PNGase F are also indicated. (B) Effect of TM on the expression of GLT1D1 and glycosylation of PD‐L1. Raji cells were first treated with TM (1 µg·mL^−1^) for 48 h, and cell lysates were then analyzed by western blot. (C) Effect of TM on GLT1D1 mRNA expression in Raji cells. GLT1D1 mRNA was analyzed by qRT‐PCR in triplicate. Bars, means ± SD; NS, no statistical significance (*P* > 0.05, Student's *t*‐test). (D) Protein lysates from Raji cells pre‐treated with or without TM were digested with PNGase F and other deglycosylases. The products were analyzed by western blotting using GLT1D1 antibody. (E) Schematic model of GLT1D1‐mediated N‐glycosylation of PD‐L1. GLT1D1 consists of a short cytoplasmic N‐terminal, a single transmembrane domain (TMD), a ‘stem’ region, and a catalytic domain (Heinonen *et al.*, 2003; Helenius and Aebi, 2001). GLT1D1 function as a glycosyltransferase, which could mediate the transfer of N‐glycans to the Asn residue of PD‐L1.

In mammalian cells, the N‐linked glycosylation of proteins follows a highly conserved pathway (Helenius and Aebi, 2001). As shown in Fig. 2A, a 14‐saccharide core unit (Glc3Man9GlcNAc2) is first assembled as a membrane‐bound dolichylpyrophosphate (Dol‐PP) precursor, which is then further processed in the ER for transferring to the nascent polypeptide chains. A main function of GLT1D1 is to act as a glycosyltransferase that transfers glycosyl groups such as galactose, *N*‐acetylglucosamine, and sialic acid to proteins through the formation of glycosidic bonds. Since N‐linked glycosylation (N‐glycosylation) of PD‐L1 is essential for maintaining PD‐L1 protein stability and its immune checkpoint function, we asked whether GLT1D1 promoted PD‐L1 N‐glycosylation in B‐cell NHL cells. We first used tunicamycin (TM), an N‐glycosylation inhibitor known to block the reaction between UDP‐*N*‐acetylglucosamine and dolichol phosphate in the early step of glycoprotein synthesis (Inukai *et al.*, 1993), to test its effect on GLT1D1 and PD‐L1 in Raji cells. Notably, a reduction in the molecular weight of GLT1D1 and a parallel conversion of high‐molecular‐weight PD‐L1 protein to lower molecular weight were detected after Raji cells were treated with TM for 48 h (Fig. 2B) without any change in GLT1D1 mRNA (Fig. 2C), suggesting a suppression of GLT1D1 binding to its glycosyl‐substrate and a reduction of glycosylated PD‐L1 by TM treatment. We then used a enzymatic protein deglycosylation Kit (Sigma‐Aldrich) containing the key enzymes (PNGase F and other deglycosylases) and reagents needed to remove all N‐linked and simple O‐linked carbohydrates from glycoproteins to further evaluate the ability of GLT1D1 to modify PD‐L1 through N‐glycosylation. After treatment of cell lysates with PNGase F (an enzyme that effectively remove most N‐linked oligosaccharides from glycoproteins) and/or other deglycosylases, no significant changes of GLT1D1 were detected (Fig. 2D), whereas treatment of cells with TM consistently caused a shift of the GLT1D1 band to lower molecular weight (Fig. 2D). These data together indicated that GLT1D1 protein *per se* was not N‐glycosylated and that the high‐molecular‐weight band was most likely the substrate‐bound GLT1D1, whereas the low‐molecular‐weight band was the enzyme without binding to its substrate, which was depleted when cells were treated with TM.

We also used fetuin, a glycoprotein containing sialylated N‐linked and O‐linked glycans, as a positive control for the PNGase F enzyme activity, using an assay kit from New England BioLabs (Ipswich, MA, USA). Incubation of fetuin with PNGase F caused an almost complete cleavage of the N‐linked oligosaccharides, thus shifting the fetuin band to a lower molecular weight position (Fig. S1A). In contrast, incubation of Raji cell lysates with PNGase F did not cause any shift of the GLT1D1 band (Fig. S1B). These results support the conclusion that GLT1D1 protein itself was not N‐glycosylated, but it could mediate PD‐L1 N‐glycosylation by transferring its bound substrate (N‐glycan) to PD‐L1, as illustrated in Fig. 2E. Using GC/MS‐based analysis, we found that the relative abundance of total mannose from biomass‐bounded glycan significantly decreased after GLT1D1 knockdown (Fig. S2), indicating that the glycan profile was severely disrupted when GLT1D1 was silenced.

**Fig. 3:**
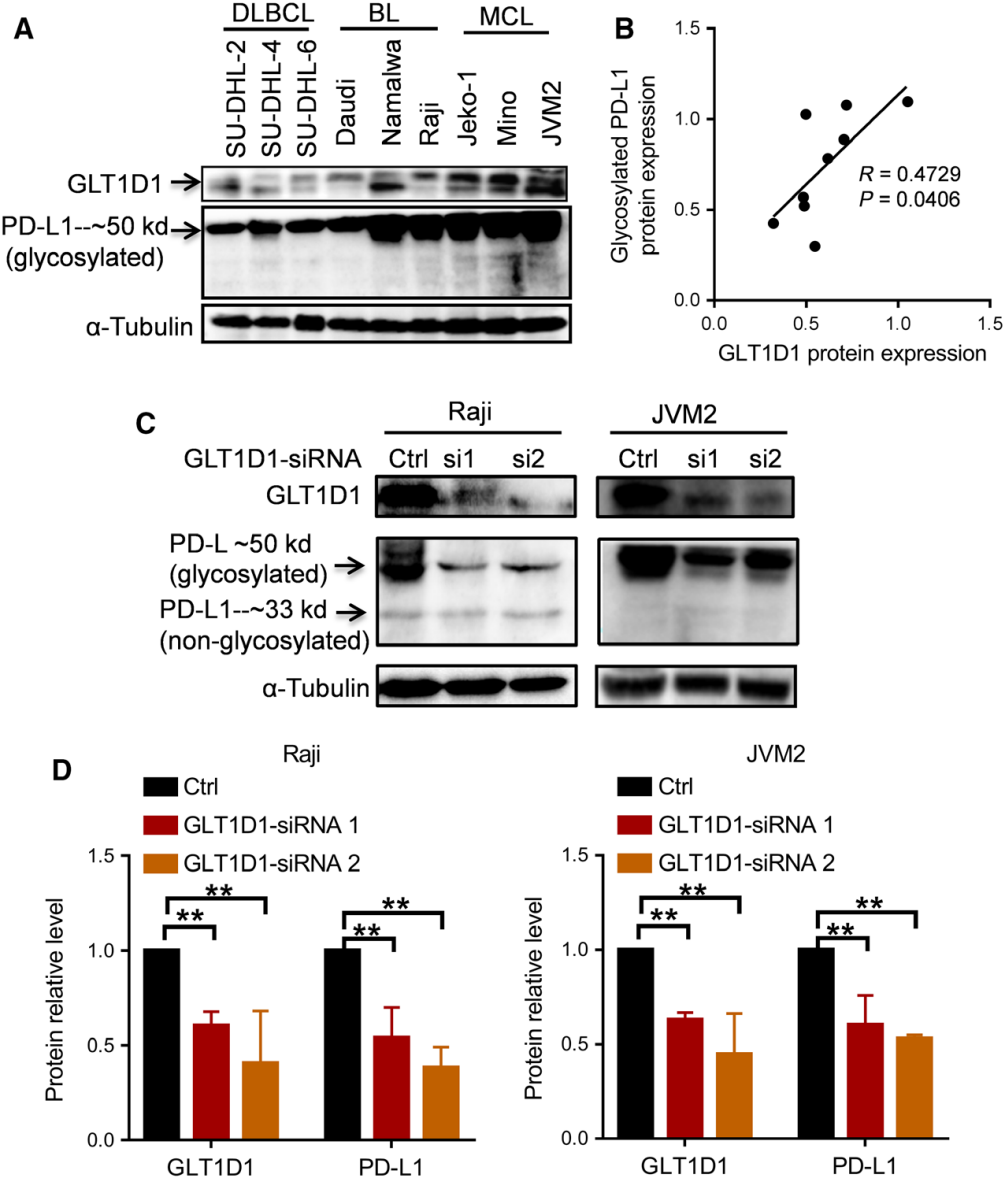
Role of GLT1D1 in glycosylation of PD‐L1. (A) Western blot analysis of glycosylated PD‐L1 and GLT1D1 expression in nine lymphoma cell lines representing three subtypes (DLBCL, BL, MCL) of B‐cell NHLs. (B) Correlation between GLT1D1 protein expression and the level of glycosylated PD‐L1 in B‐cell NHLs (Pearson's correlation analysis). (C) Knockdown of GLT1D1 expression led to a major decrease of PD‐L1 glycosylation in Raji cells and JVM2 cells, detected by western blot analysis. (D) Quantitation of GLT1D1 protein expression and glycosylated PD‐L1 and in Raji (left panel) and JVM2 (right panel) cells after siRNA silencing of GLT1D1. Data were from two experiments with two replicates each. Bars, means ± SD; **P < 0.01 (one‐way ANOVA).

Considering the facts that glycosylation of PD‐L1 by N‐glycosyltransferease can stabilize PD‐L1 protein and that GLT1D1 expression is associated with poor prognosis in NHLs, we investigated whether GLT1D1 could induce immunosuppression through glycosylation and stabilization of PD‐L1 in B‐cell NHLs. Western blot analysis showed that glycosylation of PD‐L1 was positively correlated with GLT1D1 protein level (Fig. 3A,B). To test the cause–effect relationship between GLT1D1 expression and glycosylation of PD‐L1, we transfected B‐cell lymphoma lines (Raji and JVM2) with relatively high level of GLT1D1 with siRNA against GLT1D1 and then analyzed glycosylated PD‐L1. As shown in Fig. 3C,D, knocking down of GLT1D1 significantly decreased the level of glycosylated PD‐L1 protein. In contrast, silencing of GLT1D1 did not change the level of nonglycosylated PD‐L1 protein, which was unstable and appeared only as a light band around 33 kD (Fig. 3C).

### 3.3 High GLT1D1 expression is correlated with glycosylated PD‐L1 in DLBCL tissues and is associated with poor prognosis

**Fig. 4:**
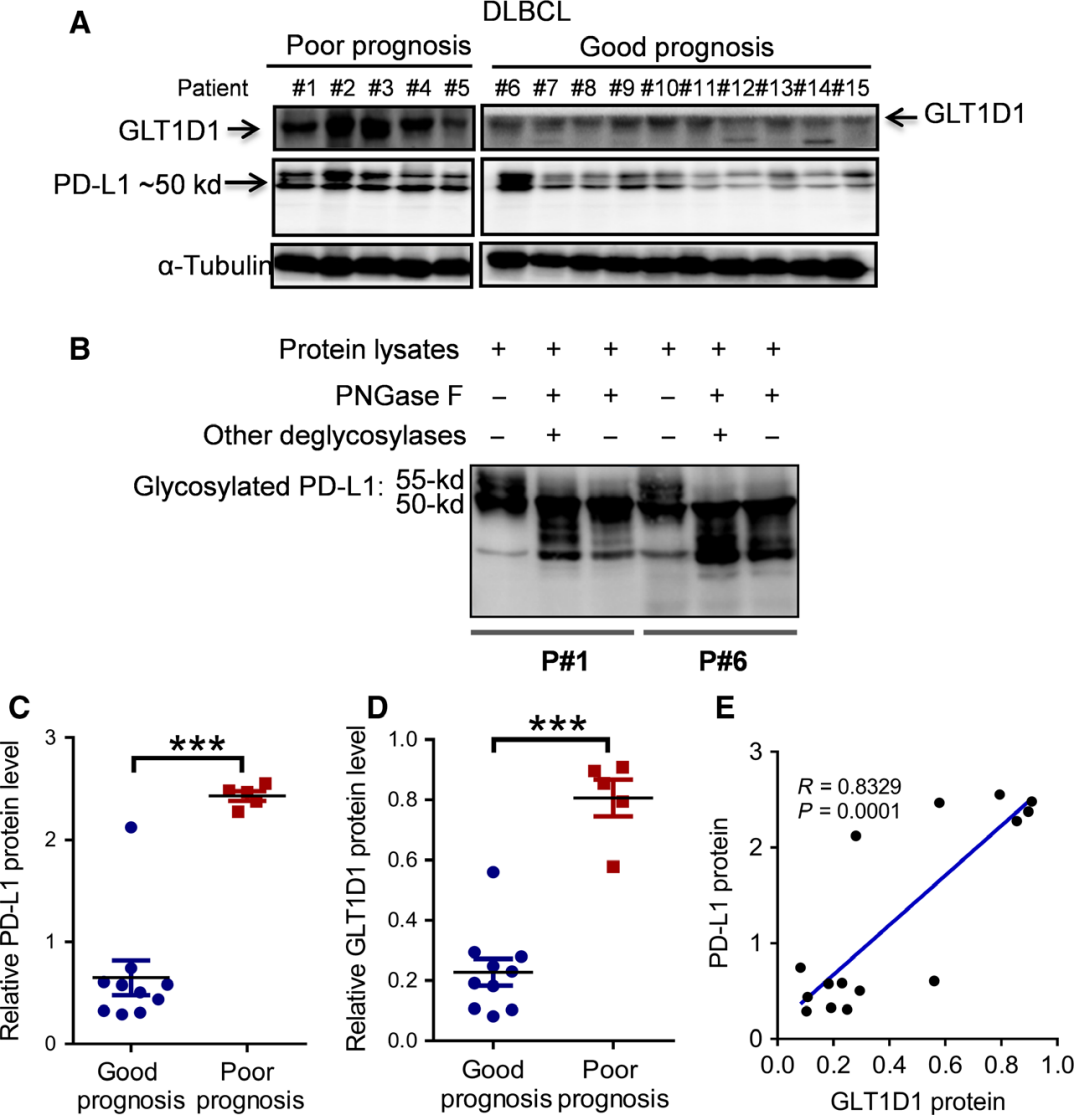
Relationship between GLT1D1 protein expression and PD‐L1 glycosylation in clinical specimens of DLBCL patients with good or poor prognosis. (A) Western blot analysis of GLT1D1 expression the glycosylated PD‐L1 in lymphoma samples of DLBCL patients with poor prognosis (*n* = 5) or good prognosis (*n* = 10). (B) Analysis of glycosylation patterns of PD‐L1 protein in DLBCL specimens by digestion of cell lysates with different deglycosylases. Protein lysates of two patient samples were treated with the indicated enzymes from Protein Deglycosylation Kit (‘other deglycosylases’ included F+a‐(2→3,6,8,9)‐neuraminidase, O‐glycosidase, β‐N‐acetyl‐glucosaminidase, β‐(1→4)‐galactosidase), and the digestion products were analyzed by western blotting. (C) Quantitative comparison of glycosylated PD‐L1 in DLBCL samples from patients with good or poor prognosis (Student's *t*‐test). (D) Quantitation of GLT1D1 protein expression in DLBCL samples from patients with good or poor prognosis (Student's *t*‐test). (E) Correlation analysis of GLT1D1 protein expression and the levels of glycosylated PD‐L1 in DLBCL patient samples. Pearson's correlation analysis was used, *n* = 15. Bar, mean ± SD; ***P < 0.001.

To further evaluate the relationship between GLT1D1 and PD‐L1 in human DLBCL patients, we analyzed the correlation between GLT1D1 expression and glycosylated PD‐L1 in lymphoma patient tissues. GLT1D1 and glycosylated PD‐L1 protein levels were analyzed in the tumor tissues from 15 DLBCL patients (5 patients with poor prognosis and 10 patients with good prognosis). As shown in Fig. 4A and Fig. S3, both GLT1D1 and glycosylated PD‐L1 protein levels were higher in the patient samples with poor prognosis compared to those with good prognosis. Interestingly, in addition to the glycosylated PD‐L1 observed at 50 kD, a ~55 kD band was also detected. Digestion of the protein lysates with deglycosylation enzymes caused the 55‐kD band to shift to lower molecular weights (Fig. 4B), suggesting that it was a different form of glycosylated PD‐L1 protein. Quantitative analysis of the protein band density showed that the levels of GLT1D1 and glycosylated PD‐L1 were significantly higher in patients with poor prognosis compared to those with good prognosis (Fig. 4C,D). A strong correlation between the glycosylated PD‐L1 levels and GLT1D1 protein was observed (*P* < 0.0001, Fig. 4E).

### 3.4 GLT1D1‐induced glycosylation of PD‐L1 impairs T‐cell function

**Fig. 5:**
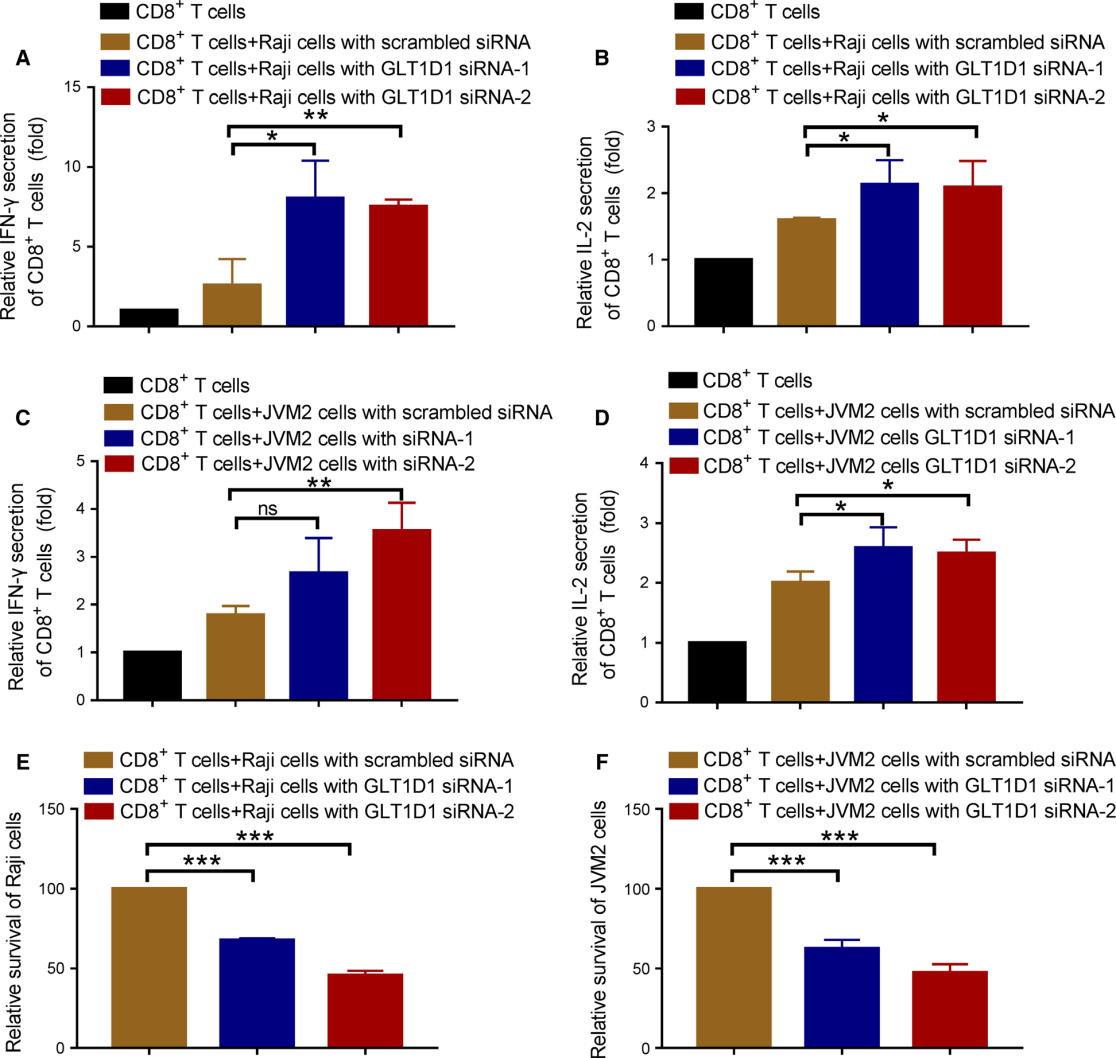
Impact of silencing GLT1D1 in lymphoma cells on T‐cell functions in coculture system. (A) IFN‐γ production by activated CD8^+^ T cells cocultured with Raji cells transfected with GLT1D1‐siRNA or scrambled siRNA as described Materials and methods. IFN‐γ secretion was measured by ELISA. (B) IL‐2 production by activated CD8^+^ T cells cocultured with Raji cells transfected with GLT1D1‐siRNA or scrambled siRNA. IL‐2 secretion was measured by ELISA. (C) IFN‐γ production by activated CD8^+^ T cells cocultured with JVM2 cells transfected with GLT1D1‐siRNA or scrambled siRNA. IFN‐γ secretion was measured by ELISA. (D) IL‐2 production by activated CD8^+^ T cells cocultured with JVM2 cells transfected with GLT1D1‐siRNA or scrambled siRNA. IL‐2 secretion was measured by ELISA. (E) Raji cells transfected with GLT1D1‐siRNA or with scrambled siRNA were cocultured with activated CD8^+^ T cells for 72 h, and loss of cell viability was determined by measuring the release of LDH. (F) JVM2 cells transfected with GLT1D1‐siRNA or with scrambled siRNA were cocultured with activated CD8^+^ T cells for 72 h, and loss of cell viability was determined by measuring release of LDH. Bar, mean ± SD; NS, no statistical significance; *P < 0.05, **P < 0.01, ***P < 0.001 (one‐way ANOVA).

As PD‐L1 glycosylation is essential for its stabilization and modulation of immune function, we reasoned that GLT1D1 could induce cancer immunosuppression by maintaining PD‐L1 protein glycosylation and stabilization. A coculture system was used to test the effect of siRNA knockdown of GLT1D1 in B‐cell lymphoma on the functions of activated T cells. The functions of T cells were measured by quantifying the secretion of IFN‐γ and IL‐2, and by testing their ability to kill tumor cells with the release of LDH. As shown in Fig. 5A,B, coculture of activated CD8^+^ T lymphocytes with GLT1D1‐knockdown Raji cells showed a significant higher IFN‐γ and IL‐2 production compared with coculture of activated CD8^+^ T lymphocytes and the control Raji cells. Consistent results were obtained with another coculture system using activated CD8^+^ T lymphocytes and B‐cell NHL line (JVM2) with or without GLT1D1 knockdown (Fig. 5C,D). The knockdown of GLT1D1 led to a significant increase of cytotoxic T‐cell function, as evidenced by a significant loss cell viability in both coculture systems (Fig. 5E, F).

### 3.5 GLT1D1 is required for cell survival *in vitro* and tumorigenesis *in vivo*

**Fig. 6:**
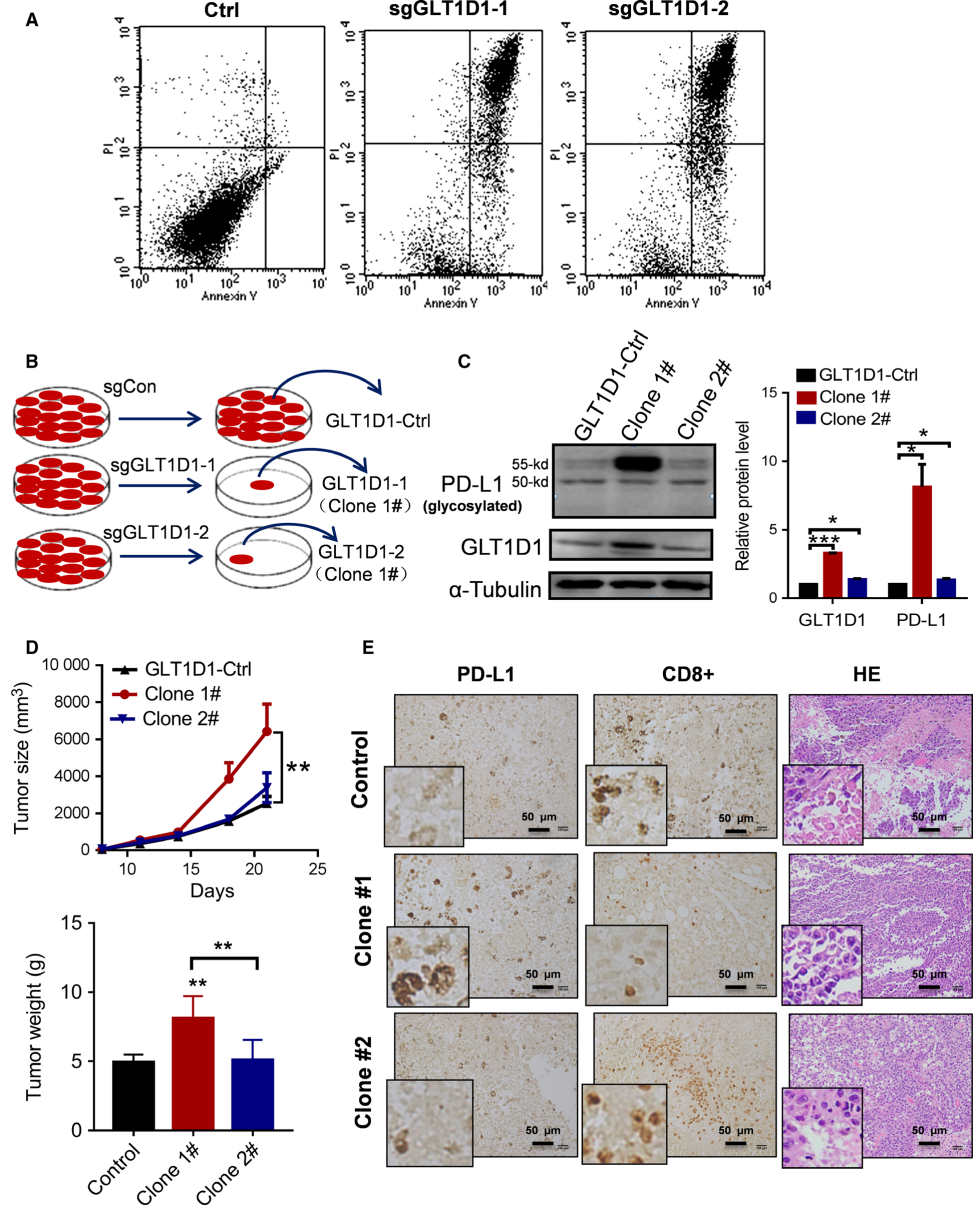
The role of GLT1D1 in affecting cell survival *in vitro* and tumorigenesis *in vivo*. (A) Impact of GLT1D1 knockout on cell viability. Lymphoma A20 cells were subjected to CRISPR/Cas9‐mediated knockout with gRNA specific to GLT1D1 or with control gRNA as described in Materials & Methods. Cell viability was analyzed by flow cytometry using annexin‐V/PI staining. (B) Melanoma B16 cells were subjected to CRISPR/Cas9‐mediated knockout with gRNA specific to GLT1D1 or with control gRNA. After selection, two clones of survival cells were isolated for comparison with the control cells. (C) Comparison of PD‐L1 and GLT1D1 protein expression in B16 control cells and the two selected clones. Representative western blots were shown in left panel, and quantitative protein levels of GLT1D1 and glycosylated PD‐L1 were shown in right panel (one‐way ANOVA). (D) Tumor sizes and tumor weights of B16 xenografts from mice inoculated with control B16 cells or the two selected B16 clones (*n* = 5 mice per group) were compared using two‐way and one‐way ANOVA analyses, respectively. (E) Immunohistochemical staining for PD‐L1, CD8^+^T cells in tumor tissues isolated from mice inoculated with control B16 cells or the two selected B16 clones. The tissues were also stained with H&E. The image at the low‐left corner of each panel shows the higher magnification of the representative image. The scale bar indicates 100 μm, Bars, means ± SD. *P < 0.05, **P < 0.01, ***P < 0.001.

We next evaluated the role of GLT1D1 in maintaining cell viability *in vitro* and tumor growth *in vivo*. To this end, we first used CRISPR‐Cas9 technology to knockout GLT1D1 in mouse B‐cell lymphoma cells (A20 cell line) and tested cell survival in culture and tumor formation *in vivo*. We were unable to recover any viable GLT1D1‐knockout A20 cells, since the sgGLT1D1‐targeted A20 cells lost their ability to survive in culture (Fig. 6A), suggesting GLT1D1 might be required for the survival of the lymphoma cells. We then used mouse B16 melanoma cells as an alternative model for the GLT1D1 knockout study. After CRISPR/Cas9‐mediated disruption of GLT1D1, a small fraction (1–2%) of the sgGLT1D1‐targeted cells survived (Fig. 6B). Two clones of the survival cells were successfully isolated and expanded for further study. However, analysis of GLT1D1 expression by western blotting showed that both survival clones still expressed GLT1D1 protein, with Clone #1 unexpectedly overexpressed a higher level of GLT1D1 (Fig. 6C). These results support the notion that GLT1D1 might be essential for cell survival, just like that observed in the lymphoma cells.

**Fig. 7:**
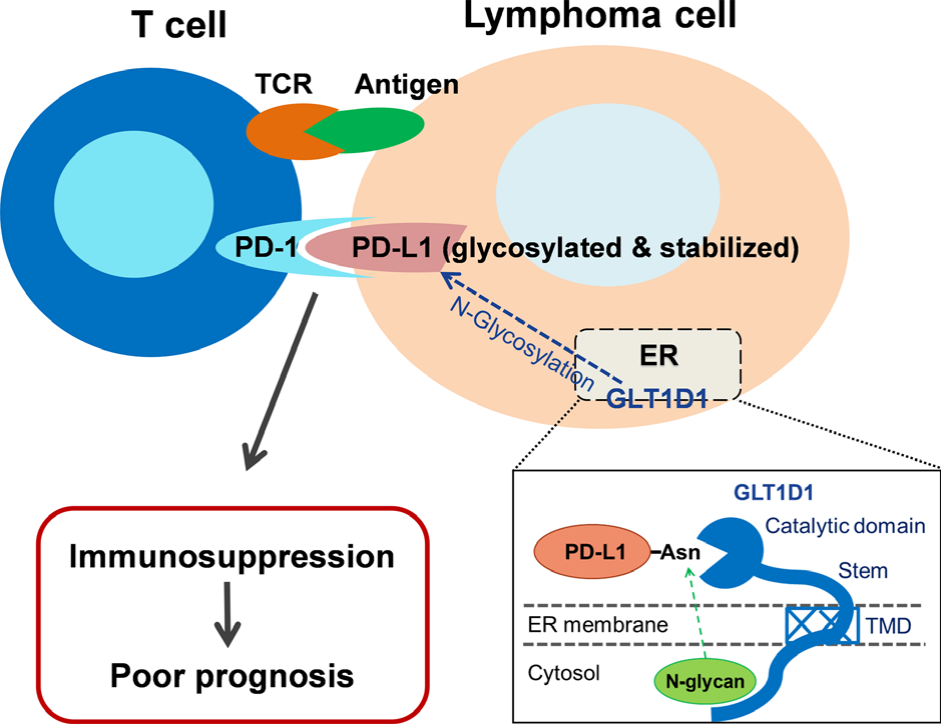
Schematic diagram showing the role of GLT1D1 in mediating PD‐L1 glycosylation and the subsequent impact on clinical outcome. The high expression of GLT1D1 in B‐cell lymphoma promotes the transfer of N‐glycans to the asparagine (Asn) residue of PD‐L1. The N‐glycosylation of PD‐L1 enhances its stability and thus promotes the interaction between PD‐L1 on the lymphoma cells and PD‐1 on the T cells, leading to immune suppression and poor clinical outcome.

The elevated GLT1D1 expression in Clone #1 could be due to a CRISPR/Cas9‐mediated gene alteration leading to an overexpression variant. The two clones of GLT1D1‐expressing B16 cells were expanded and inoculated into syngenic C57BL mice to evaluate their capacity to form tumor *in vivo.* One week after inoculation, tumor sizes were measured every 3 days. The experiment was terminated at the end of 3 weeks, and tumors were removed for immunohistochemistry staining for PD‐L1 expression and CD8^+^ T‐cell infiltration. Notably, clone 1# xenograft, which expressed high level of GLT1D1, exhibited a significant increase in tumor growth (Fig. 6D), associated with a high level of PD‐L1 expression and very few CD8^+^ T‐cell infiltration in the tumor tissues (Fig. 6E). Clone #2, which expressed a similar level of GLT1D1 as the parental cells, showed similar tumor growth (Fig. 6D) and comparable PD‐L1 expression and CD8^+^ T‐cell infiltration as the control xenograft (Fig. 6E). These data together suggest that GLT1D1 could promote PD‐L1 glycosylation and enhance its stabilization *in vivo*, and thus promote tumor growth by suppressing cytotoxic CD8^+^ T cells in the tumor microenvironment, as illustrated schematically in Fig. 7.

## 4 Discussion

Non‐Hodgkin's lymphoma is a heterogeneous disease, and patients with relapsed or refractory NHL have little therapeutic options and generously have poor prognosis. Immunosuppression is one of the important contributing factors for poor clinical outcome of NHLs (Chen *et al.*, 2013; Georgiou *et al.*, 2016; Kiyasu *et al.*, 2015; Rossille *et al.*, 2014). Thus, immunotherapy represents a promising new therapeutic strategy for treatment of NHLs and could significantly improve the patient outcome. Since the clinical use of the anti‐CD20 monoclonal antibody rituximab in the treatment of lymphoma in 1997, several novel immunotherapy strategies harnessing the ability of T cells to target cancer cells have emerged. The immune checkpoint inhibitors that target PD‐1 have shown promising efficacy in lymphoma, especially in HL. However, in most of B‐NHL malignancies, the clinical response to immune checkpoint inhibitors has not been satisfactory, and many patients do not receive durable benefit, suggesting an urgent need to better understand the molecular and genetic mechanisms that influence lymphoma response to immunotherapy. Recent studies demonstrated that the N‐linked glycosylation of PD‐L1 is important for stabilizing PD‐L1 protein. In this study, through comparing the highly curable DLBCL and the incurable MCL cells, or comparing DLBCL patients with good prognosis and poor prognosis for their expression of genes involved protein glycosylation, we found that GLT1D1 expression was highly expressed in the incurable MCL cells and in poor prognosis DLBCL patients who relapsed within 6 months of initial treatment. Mechanistically, we showed that GLT1D1 played a major role in N‐glycosylation and stability of PD‐L1 protein. Downregulation of GLT1D1 caused a decrease in glycosylated PD‐L1 protein, leading to an increase in cytotoxic T‐cell infiltration in the tumor microenvironment.

PD‐L1 is an immunosuppressive molecule, which is expressed on a variety of carcinoma cells and inflammatory‐activated immune cells. PD‐L1 on cancer cells or on the tumor microenvironment cells interacts with PD‐1 on T cells, inducing immune cells exhaustion and immune escape. Recent study has revealed that glycosylation is required for the PD‐L1/PD‐1 interaction and is important for its immunosuppressive function, and that glycotransferase B3GNT3 catalyzes such glycosylation in triple‐negative breast cancer (Li *et al*, 2018). In our study, we found that glycotransferase GLT1D1 plays an important role in N‐glycosylation of PD‐L1 in B‐NHLs. We showed that GLT1D1 was significantly upregulated in incurable lymphoma (MCL) and in a subset of DLBCL patients with poor prognosis, suggesting that high expression of GLT1D1 may predict high risk of lymphoma relapse, likely due in part to immunosuppression. Indeed, several studies suggested that high expression of PD‐L1 was associated with poor OS in lymphoma (Chen *et al.*, 2013; Georgiou *et al.*, 2016; Kiyasu *et al.*, 2015; Rossille *et al.*, 2014). Our data analysis also revealed that GLT1D1 was positively correlated with PD‐L1 expression in lymphoma. It should be noted, however, that a high expression of GLT1D1 with high PD‐L1 N‐glycosylation may not always predict poor prognosis. There could be exceptions. For instance, we noticed that among the 10 samples from good prognosis patients, one of them expressed relatively high level of GLT1D1 with a highly elevated PD‐L1 N‐glycosylation similar to that of the poor prognosis samples (Fig. 4A). A possible explanation is that the prognosis of lymphoma patients could be affected by multiple factors. Although PD‐L1 could impact tumor immune response and thus influence the clinical outcome, other factors that regulate cancer cell growth, metastasis, and drug sensitivity could also affect the patient prognosis.

In addition to B3GNT3 in breast cancer and GLT1D1 in NHLs that affect glycosylation of PD‐L1, other glycosyltransferase enzymes such as GCNT3 have also been shown to impact the clinical outcome of colon and ovarian cancers, and are considered as a prognostic biomarker for the identification of early‐stage colon cancer patients at high risk of relapse (Fernandez *et al.*, 2018; Gonzalez‐Vallinas *et al.*, 2015). It is unclear whether GCNT3 could also affect PD‐L1 glycosylation and modulate immunocheckpoint. Protein glycosylation could also impact cancer cells by affecting their interaction with galectins (Fernandez *et al.*, 2018; Gonzalez‐Vallinas *et al.*, 2015), and by stabilizing GRP78 protein through O‐glycosylation (Lin *et al.*, 2017). All these observations together indicate that glycosyltransferases could be used as potential biomarkers for predicting clinical outcome of cancer patients.

Glycosyltransferases could be deregulated at multiple levels, including epigenetics, transcription, post‐transcription, and/or changes in chaperone. Altered glycosidase activity, altered expression of glycoconjugate acceptor together with availability and abundance of the sugar nucleotide donors, and altered sugar nucleotide transporter activity could also affect glycosylation (Padler‐Karavani, 2014). The mechanisms by which glycosyltransferases are upregulated in cancer remain unclear at the present time. For increased glycosylation of PD‐L1 protein, it is unclear whether the upregulation of a particular glycosyltransferase is cancer type‐dependent. Obviously, further studies in this area are needed.

Cytotoxic CD8^+^ T cells have been found to associate with a Th1‐oriented immune reaction and are strongly correlated with good clinical outcome in multiple cancer types (Fridman *et al.*, 2012). Reversing the immunosuppressive tumor environment by blocking PD‐L1 expression could release the inhibitory effect on cytotoxic CD8^+^ T cells (Tao *et al.*, 2019). These might partially explain our findings that low infiltrating CD8^+ ^T cells in the tumor xenografts of Clone 1# cells, which expressed very high levels of GLT1D1 and glycosylated PD‐L1. Upregulation of GLT1D1 helps tumors escape from the immune surveillance by stabilizing PD‐L1 protein through increased glycosylation of PD‐L1, which interacts with PD‐1 on T‐cell surface and negatively impact T‐cell viability and function (Fig. 7).

## 5 Conclusions

In summary, we found that upregulation of GLT1D1 was strongly correlated with early relapse and associated with poor prognosis in B‐NHL. GLT1D1 played a major role in enhancing PD‐L1 stability through N‐glycosylation. Downregulation of GLT1D1 decreased the glycosylated PD‐L1 protein and promoted cytotoxic T‐cell function against lymphoma cells. As such, targeting GLT1D1 could be a novel strategy to combat PD‐L1/PD‐1 interaction‐mediated immunosuppression in B‐NHLs and could potentially increase the clinical outcome of lymphoma patients.

## Conflict of interest

The authors declare no conflict of interest.

## Author contributions

PL, PH, and XL performed the conception and design. XL, YZ, YH, WL, JT, TY, and JY involved in the acquisition of data. XL, YZ, YH, WL, PL, PS, and HZ involved in the analysis and interpretation of data. XL, PH, and PL wrote, reviewed, and/or revised the manuscript. YH and YZ involved in the study supervision. All authors read and approved the final manuscript.

## Ethics approval and consent to participate

The use of lymphoma specimens was approved by the Institutional Research Ethics Committee of Sun Yat‐sen University Cancer Center. All animal experiments were performed in accordance with a protocol approved by the ethics committee of the Institutional Animal Care of Sun Yat‐sen University Cancer Center.

## Supporting information


**Fig. S1. **Deglycosylation of glycoproteins by PNGase F.
**Fig. S2. **The relative level of mannose in Raji cells before and after knockdown of GLT1D1 expression by siRNA.
**Fig. S3. **Original western blots of Fig. 4A.

**Table S1. **The primers and sequences for qRT‐PCR array.

## Data Availability

The dataset used and/or analyzed during the current study are available from the corresponding author on reasonable request.
