# Supplementary material for: Overexpression of GLT1D1 induces immunosuppression through glycosylation of PD‐L1 and predicts poor prognosis in B‐cell lymphoma
Source: Mol Oncol. 2020 Apr 13;14(5):1028–44. doi: 10.1002/1878-0261.12664 (PMC7191186; doi:10.1002/1878-0261.12664)
Supplement: Supplementary file 1 — Fig. S1. Deglycosylation of glycoproteins by PNGase F. Fig. S2. The relative level of mannose in Raji cells before and after knockdown of GLT1D1 expression by siRNA. Fig. S3. Original western blots of Fig. 4A. [file MOL2-14-1028-s001.docx]

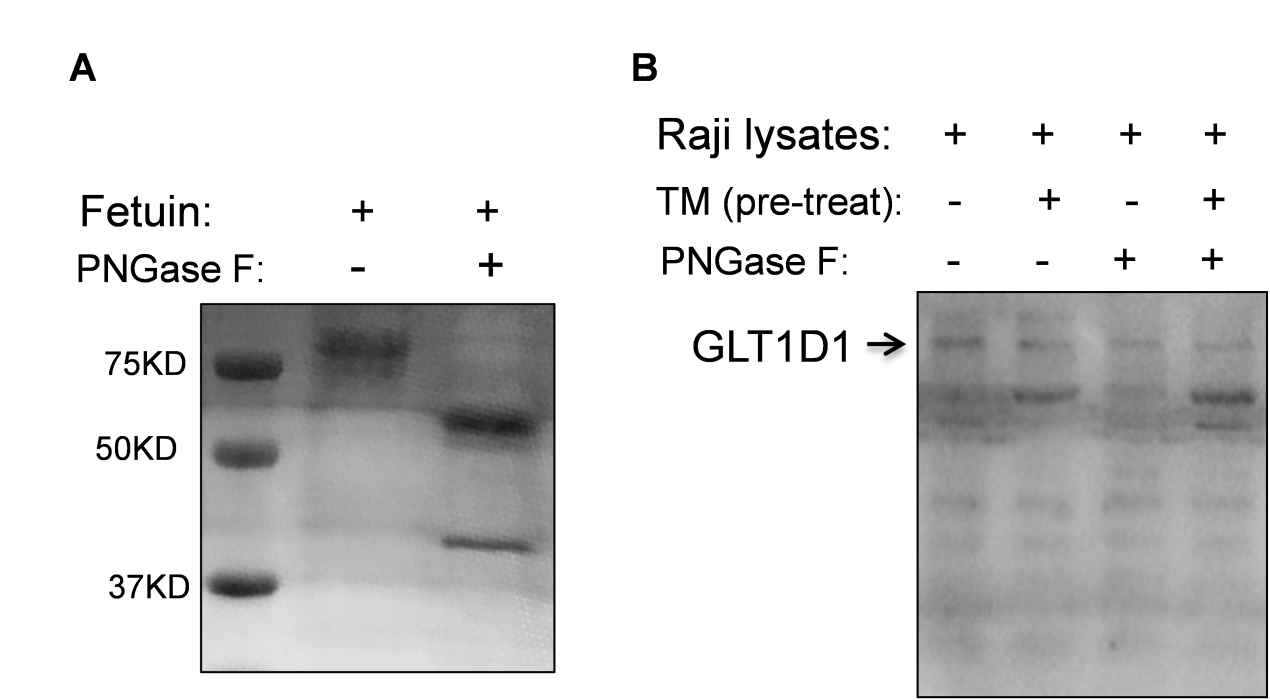


**Fig. S1.** Deglycosylation of glycoproteins by PNGase F. **A.** Analysis of deglycosylation of fetuin by incubation with PNGase F. The digestion products were analyzed on a 10% SDS-PAGE, followed by staining with Coomassie blue. **B**. Cell lysates from Raji cells pre-treated with or without tunicamycin (TM) as were incubated with PNGase F as indicated. The digestion products were analyzed on a 12% SDS-PAGE followed by western blotting using GLT1D1 antibody.


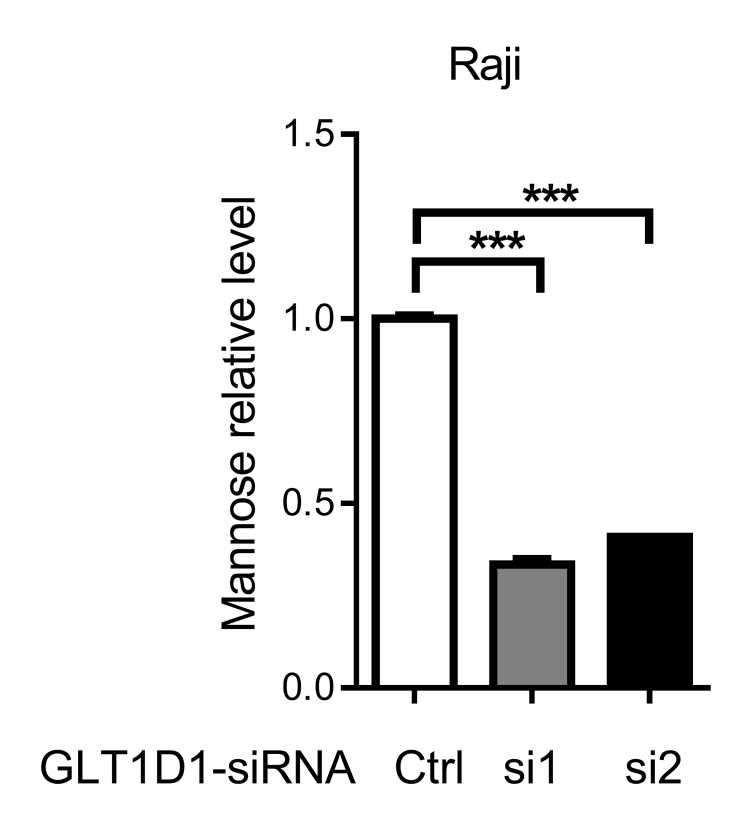


**Fig. S2.** The relative level of mannose in Raji cells before and after knockdown of GLT1D1 expression by siRNA. Mannose was measured by GC-MS analysis. The relative level of mannose was normalized by the protein contents of the respective samples. Bars, means ± SD. ****p* < 0.001 (One-way ANOVA).


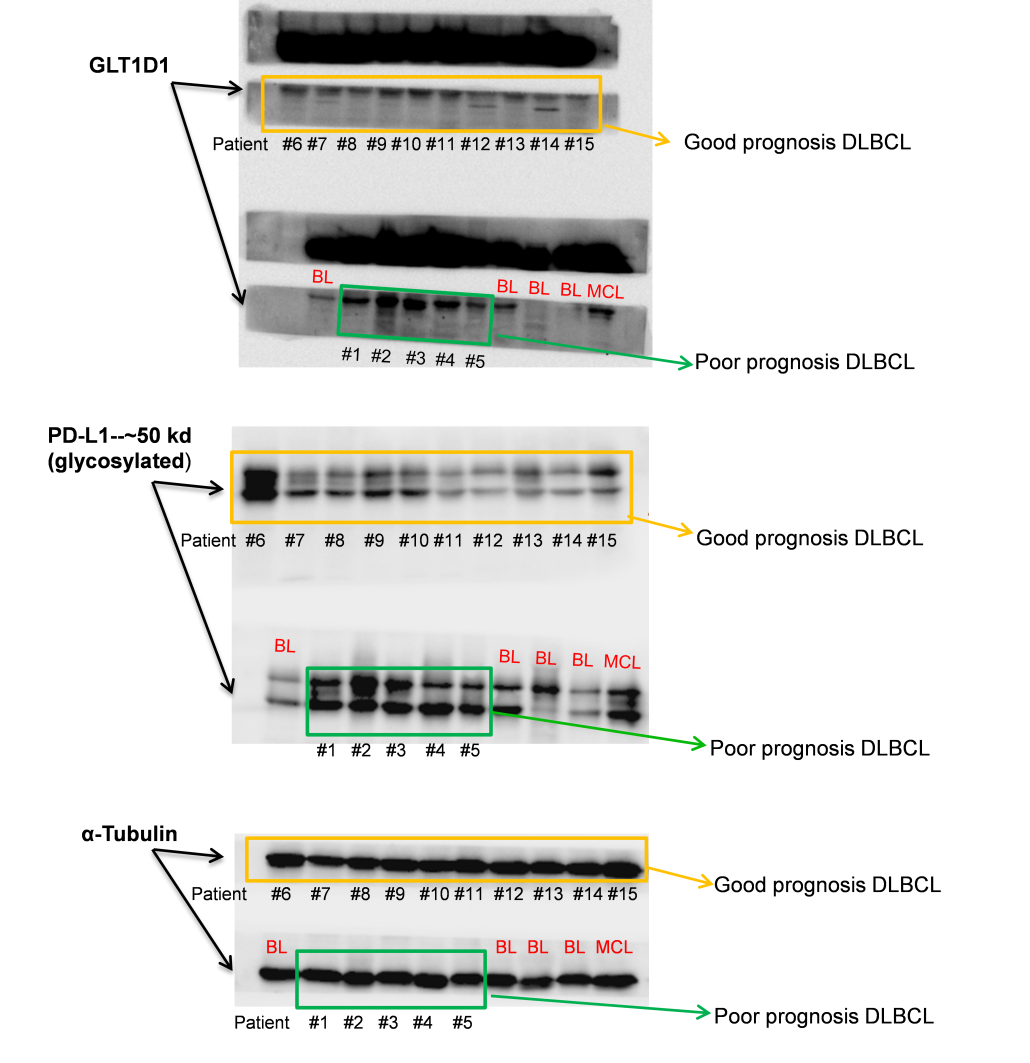


**Fig. S3.** Original western blots of Fig. 4A. The areas in color boxes are all diffuse large B cell lymphoma (DLBCL) samples shown in Fig. 4A; the lanes marked in red “BL” are Burkit lymphoma samples with good prognosis; the lane marked in red “MCL” is a sample from mantle cell lymphoma with poor prognosis.
