# Supplementary material for: Overexpression of GLT1D1 induces immunosuppression through glycosylation of PD‐L1 and predicts poor prognosis in B‐cell lymphoma
Source: Mol Oncol. 2020 Apr 13;14(5):1028–44. doi: 10.1002/1878-0261.12664 (PMC7191186; doi:10.1002/1878-0261.12664)
Supplement: Supplementary file 2 — Table S1. The primers and sequences for qRT‐PCR array. [file MOL2-14-1028-s002.docx]

**Table S1: The primers and sequences for qRT-PCR array.**

| Primer Name | Sequence(5'to3') |
| --- | --- |
| GLT1D1-F | CCGCTGGGGTACGATTGATT |
| GLT1D1-R | TGTGACTTCATGCTTCACCAC |
| MGAT3-F | ATGAAGATGAGACGCTACAAGC |
| MGAT3-R | GCTGGACACCAGGTTAGGG |
| B3GNT3-F | TCCTCCTCTTCAGTCTGCTAGT |
| B3GNT3-R | CCGGGTGGGTGACCATAGA |
| GNPTAB-F | AATGGAGCCGAGATCAATACCA |
| GNPTAB-R | CTCTGACCTGCTGTAGTTCCT |
| ST6GAL1-F | AACTCTCAGTTGGTTACCACAGA |
| ST6GAL1-R | GGTGCAGCTTACGATAAGTCTT |
| MAN2B1-F | TGCAGCCGAACATGCTGAA |
| MAN2B1-R | CACGTAAATGAAGCGACGGG |
| ST8SIA-F | GGCAGAGGTACAATCAGATCAGC |
| ST8SIA-R | ATGTCTCCATTTGGACGAGGC |
| UGGT1-F | GCCTTCCAGCAGATAGCAGC |
| UGGT1-R | GCTTTCAGGATTAGACGAGGGAT |
| ST8SIA4-F | ATGCGCTCCATTAGGAAGAGG |
| ST8SIA4-R | GAGCTATTGACAAGTGACCGAC |
| B4GALT2-F | GGGCAGACTGCTGATCGAG |
| B4GALT2-R | CCGGTGTCTAAAGGGGATGAT |
| MGAT5-F | TCTGCACTTTACCATCCAGCA |
| MGAT5-R | CCAATGCGCTGCAAAATGTTAT |
| MOGS-F | CCGGGGACTCCTAAGCTCA |
| MOGS-R | CCTCTTGACGAACTCAGTGGT |
| MGAT1-F | CCGCACCGACTTTTTCCCT |
| MGAT1-R | CCAAAGGTCATCGTTCTTGAGAT |
| MGAT5B-F | ATCCGCACAGAAGTGATGGG |
| MGAT5B-R | CAGCGATGTCGGAGACGTT |
| B4GALT1-F | GGCGTCACCCTCGTTTACTAC |
| B4GALT1-R | GGGCCAGAATCCACGACTG |
| B3GNT2-F | GGTCACGTCGGTGGTTACG |
| B3GNT2-R | GCACACTTATCCGGCTGATCTA |
| MAN1A2-F | CCAACCTTGTAGGAATACGTGG |
| MAN1A2-R | TGGTAGCACCCATTTGTGAAC |
| FUT11-F | CCGGTAGACTCCTACGGGAAA |
| FUT11-R | CATGTAGTCGTTACAGATGGCAT |
| B4GALT3-F | CGAGATCAGGGACCGACATTT |
| B4GALT3-R | GATCGTTCTGGACAGTAGGGC |
| GLB1-F | TATACTGGCTGGCTAGATCACTG |
| GLB1-R | GGCAAAATTGGTCCCACCTATAA |
| MGAT4B-F | TCGTACCTGACTGACACTCTG |
| MGAT4B-R | GGATCTCCGTGGGGAACAAG |
| GANAB-F | CCTCTCTCCATACCGAGCCTT |
| GANAB-R | TCCTGAACCGAGTCATGTTCTT |
| PRKCSH-F | ATATCCCCTCCAACCGGGTC |
| PRKCSH-R | CTTCTTCAGACGGAACCCTTC |
| MGAT4C-F | TCACCTATCGCTACCTAGCTG |
| MGAT4C-R | GGCATCACGCCAGGAAGAAT |
| NEU3-F | TCGGACCATGAACCCCTGT |
| NEU3-R | CCTCCTCAGTCAAGTCCCTCA |
| NEU4-F | GGCCACGGGATGACAGTTG |
| NEU4-R | CAGGCGGATACCCATGTGTAG |
| MAN1A1-F | TTTGATGCTGTTCAGGCTATCG |
| MAN1A1-R | CCGAGTTCAAGGTAGTGTTGG |
| ST8SIA3-F | TCGCCCTGCTGATTTTATCG |
| ST8SIA3-R | AGCGCAAATTGTGACCGGA |
| NEU2-F | CCTACGCCTACCGGAAACTTC |
| NEU2-R | CTCCCCAGTCTCGACTTCG |
| MGAT2-F | TGACAACGTCCTCGTCATCTT |
| MGAT2-R | CCTGGAAACTCGTTAGGGTACAA |
| UGGT2-F | CCTAAGCGTGCATCTCGATATG |
| UGGT2-R | CTTGCCAAGTACAACCAACAAC |
| EDEM2-F | CCCACTTCGGCCAGAACTTAT |
| EDEM2-R | CAGCTTGTGGTCTCGCAGA |
| HEXA-F | GGAGACTTTTAGCCAGCTTGT |
| HEXA-R | TGCTAGAGAGTGGCAGGTAAT |
| FUCA2-F | TTGAGGTAGCCATTAGGAACAGA |
| FUCA2-R | GCCGCTTATGGAATGAACTGG |
| MAN1B1-F | TGTCGAGATTGCAGCGGAATA |
| MAN1B1-R | GCTGGTAAGACGGGTGGATTT |
| MAN2A2-F | TGTGTGGCAGTCTTCTCGC |
| MAN2A2-R | CGGAGTCCTTGATATGGCTGAT |
| ST8SIA6-F | CGCCACTAACAGCACATATCTG |
| ST8SIA6-R | ATCTGAAGGTAGTCGTTCTCTGA |
| EDEM1-F | GCTACGACAACTACATGGCTC |
| EDEM1-R | GACTTGGACGGTGGAATCTTT |
| HEXB-F | CTCGCCCCGGAGAACTTCTA |
| HEXB-R | GAAAGCATCACACTCTGACTGA |
| MANBA-F | TGAGCTGCGTTTCCAGTCAG |
| MANBA-R | ACATGGCATTCACCCTTCTGC |
| NAGPA-F | CGACGACGACTTGCTACTG |
| NAGPA-R | TGAAGTGCGACACGAAGGTG |
| AGA-F | CGGAAGTCGAACTTGCCTGT |
| AGA-R | TCGGTTGCATTCTTAAAGGGC |
| MGAT4A-F | ACATGGTGTTGTAGCCAACCT |
| MGAT4A-R | GCCCTTTTCTTGAGCATACATCA |
| B3GNT8-F | GTCCCATTCAACCAGACGCTC |
| B3GNT8-R | GGGCACATAGAAGGGTCCTC |
| EDEM3-F | CGAGCCCATGAGTAGGGAG |
| EDEM3-R | AAAGGCATGAGTTCATCAGCA |
| NEU1-F | ACCTTGGGGCAGTAGTGAG |
| NEU1-R | TCCCGCTGTTTCTGAATACCA |
| MAN1C1-F | CCGCTTTGACTTCAACGCATT |
| MAN1C1-R | CATAACGCTTATAGCTCTGCCAA |
| FUCA1-F | GGATTTGGTTGGTGAATTGGGA |
| FUCA1-R | CATCAGACCAGATCAGATCAGGT |
| GNPTG-F | TGAGGACACTTTTTGAGGATGC |
| GNPTG-R | GTGCTGGGTGAGCAAACCTT |
| FUT8-F | GAATCTCAGAATTGGCGCTATGC |
| FUT8-R | GAAGCTCGACCACTTGAACAT |
